# Regulation of the small GTPase Rab1 function by a bacterial glucosyltransferase

**DOI:** 10.1038/s41421-018-0055-9

**Published:** 2018-10-09

**Authors:** Zhen Wang, Alix McCloskey, Sen Cheng, Mei Wu, Chenyu Xue, Zhengyou Yu, Jiaqi Fu, Yanhua Liu, Zhao-Qing Luo, Xiaoyun Liu

**Affiliations:** 1 Institute of Analytical Chemistry and Synthetic and Functional Biomolecules Center, College of Chemistry and Molecular Engineering, Peking University, Beijing, 100871 China; 2 Purdue Institute of Inflammation, Immunology and Infectious Disease and Department of Biological Sciences, Purdue University, West Lafayette, IN 47907 USA

## Abstract

Posttranslational modification of key host proteins by virulence factors is an important theme in bacterial pathogenesis. A remarkable example is the reversible modifications of the small GTPase Rab1 by multiple effectors of the bacterial pathogen *Legionella pneumophila*. Previous studies have shown that the effector SetA, dependent on a functional glucosyltransferase domain, interferes with host secretory pathways. However, the enzymatic substrate(s) of SetA in host cells remains unknown. Here, by using cross-linking mass spectrometry we uncovered Rab1 as the target of SetA during *L. pneumophila* infection. Biochemical studies establish that SetA covalently attaches a glucose moiety to Thr_75_ within the switch II region of Rab1, inhibiting its intrinsic GTPase activity. Moreover, we found that SetA preferentially modifies the GDP-bound form of Rab1 over its GTP-associated state and the modification of Rab1 inhibits its interaction with the GDP dissociation inhibitor GDI1, allowing for Rab1 activation. Our results thus add an extra layer of regulation on Rab1 activity and provide a mechanistic understanding of its inhibition of the host secretory pathways as well as cellular toxicity.

## Introduction

*Legionella pneumophila*, a gram-negative bacterium, is the etiological agent of a potentially lethal pneumonia called Legionnaires’ disease^1^. Human infections are typically associated with phagocytosis by alveolar macrophages where *L. pneumophila* resides and replicates within a membrane-bound compartment known as the *Legionella*-containing vacuole (LCV)^2^. The biogenesis of the LCV requires successful modulation of multiple host cell processes, particularly vesicle trafficking and membrane transport, which eventually leads to the formation of an organelle with features closely resembling those of the endoplasmic reticulum (ER)^3,4^. Crucial for hijacking host cellular processes, including membrane trafficking, is the Dot/Icm type IV secretion system, which delivers a large cohort of virulence factors, called effector proteins, into host cells^5,6^. By engaging in a wide variety of host cellular pathways, these effectors function to construct a niche permissive for intracellular bacterial survival and multiplication^7,8^. Therefore, functional study of these effectors as well as their roles during infection is a central theme in the field of *Legionella* pathogenesis.

*L. pneumophila* encodes more than 330 potential effector proteins, representing >10% of its proteome, which suggests that host function modulation plays an essential role in its virulence^9^. Despite extensive efforts over the years, however, <10% of these effectors have been characterized in terms of their biochemical activities and/or interacting host proteins^5,9^. In line with the maturation of the LCV into an ER-like compartment and the importance of vesicle transport between the ER and the Golgi apparatus in this process^4^, multiple Dot/Icm effectors were found to target the small GTPase Rab1^5,6^, a protein important for the initial steps in the secretory pathway^10^. For example, the transition of Rab1 between its GTP-bound active and GDP-bound inactive states is controlled by two *L. pneumophila* effectors SidM/DrrA and LepB, which function as a guanine nucleotide exchange factor (GEF) and as a GTPase activation protein (GAP), respectively^11–13^.

More intriguingly, Rab1 activity is also controlled by at least three distinct, reversible post-translational modifications catalyzed by sets of Dot/Icm effectors. First, the GX_11_DXD (x, any amino acid) adenylyltransferase domain of SidM/DrrA catalyzes AMPylation of Rab1 and locks it in the GTP-bound active form^14^. This modification is reversed by another effector SidD, which together with SidM, temporally regulates the activity of Rab1^15,16^. Strikingly, AnkX, a Fic domain-containing effector inhibits Rab1 activity by phosphorylcholination^17,18^, a process that is reversed by the dephosphorylcholinase Lem3^18^. Rather recently, we found that Rab1 is ubiquitinated by members of the SidE family effectors via a novel mechanism that does not require E1 and E2 enzymes, and such modification is regulated by SidJ that cleaves the phosphodiester bond linking phosphoribosylated ubiquitin to the substrate^19,20^.

Despite these extensive manipulations, growing evidence points to the involvement of additional *L. pneumophila* effectors in hijacking host membrane transport^21,22^. For example, in a large yeast toxicity screening performed by Isberg and co-workers, a cohort of Dot/Icm effectors were found to interfere with host vesicle trafficking, but the precise molecular mechanisms and/or host targets of these effectors were not determined^22^. One of the identified *L. pneumophila* effectors was SetA (subversion of eukaryotic vesicle trafficking A)^22^. Interestingly, SetA contains a functional glucosyltransferase domain with the typical DXD-motif (D_134_XD_136_), which was found to be essential for its toxicity in yeast and the interference of membrane transport in mammalian cells upon ectopic expression^22,23^.

Herein we set out to identify the host glucosylation target(s) of SetA. By using cross-linking high-resolution mass spectrometry, we unveiled Rab1 as the host interacting protein of SetA. Importantly, we found that during *L. pneumophila* infection, SetA directly glucosylates Thr_75_ within the switch II region of Rab1. This site is in close proximity to those attacked by AMPylation (Tyr_80_) and phosphorylcholination(Ser_79_) by SidM and AnkX, respectively^14,17,18^. Moreover, we found that glucosylation of Rab1 inhibits its GTPase activity in vitro and GDP-loaded Rab1 is a preferable substrate of SetA-catalyzed modification. Glucosylation of Rab1 inhibits its interaction with the regulatory protein GTP disassociation inhibitor 1 (GDI1), while at the same time, binding to the bacterial GEF SidM and GTP loading is not impacted.

## Results

### Small Rab GTPases were identified as potential substrates of SetA by cross-linking mass spectrometry

**Fig. 1:**
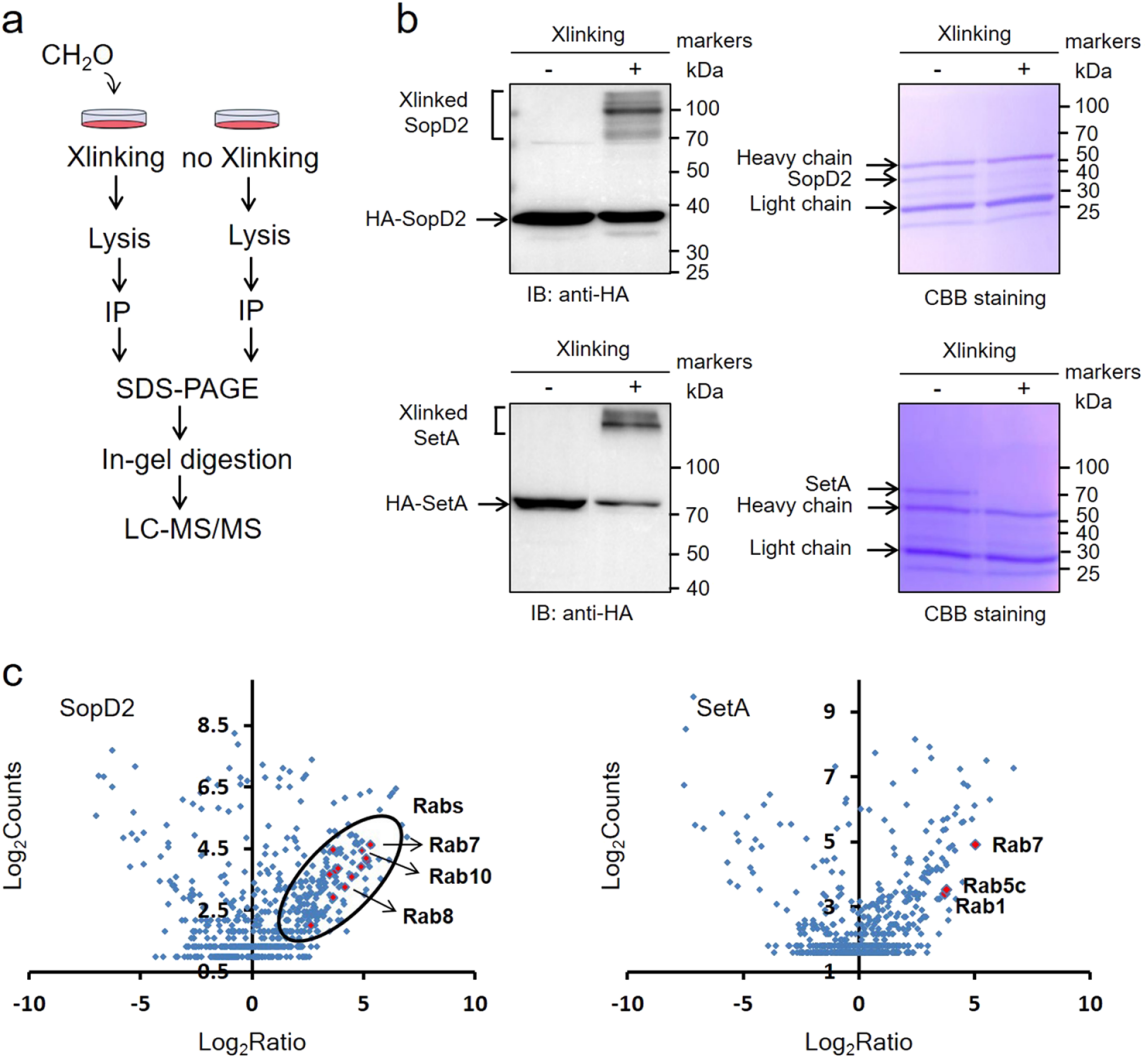
Identification of host binding proteins of bacterial effectors by a cross-linking (Xlinking) mass spectrometry strategy. **a** A schematic diagram of the overall workflow that combines in vivo formaldehyde cross-linking, affinity purification and mass spectrometry to identify SetA-interacting proteins. HEK293T cells producing HA-tagged SetA were treated with 1% formaldehyde and then lysed prior to immunoprecipitation of cross-linked protein complexes by an HA-specific antibody. The precipitates were further separated by SDS-PAGE before in-gel digestion with trypsin and LC-MS/MS analyses. **b** Monitoring of formaldehyde cross-linking reactions by immunoblotting analyses. A *Salmonella* type III effector SopD2 was included as a positive control. The Coomassie-stained gels containing cross-linked bands were processed for mass spectrometric analyses. Corresponding gel bands from non-cross-linked controls were also analyzed. **c** Scatter plots of protein ratios as a function of their relative abundance (denoted by MS/MS spectral counts). The ratio was calculated as spectral counts in cross-linked samples divided by those in non-cross-linked controls and then normalized against protein molecular weight. Large ratios indicate preferential detection in cross-linked samples, representing potential interacting substrates. Red dots correspond to detected Rab proteins in cross-linked samples

Heidtman et al. identified SetA as an *L. pneumophila* Dot/Icm substrate that inhibits yeast growth, likely by disrupting vesicle trafficking^22^. Importantly, such phenotypes were found to be strictly dependent on a predicted glycosyltransferase domain located in the N-terminus of SetA. Later, Jank et al. further established that SetA harbors mono-*O*-glucosyltransferase activity by using UDP-glucose as a sugar donor^23^. Despite these analyses, the mechanism underlying the effect of SetA expression was not known because its cellular target(s) had yet to be identified. In order to determine its eukaryotic glucosylated substrate(s), we ectopically expressed SetA in mammalian cells and analyzed the interacting host proteins by combining in vivo formaldehyde cross-linking and affinity purification-mass spectrometry (Fig. 1a). SopD2, a type III effector of *Salmonella* Typhimurium, was included as a positive control for our approach as it has been shown to interact with multiple small Rab GTPases^24–26^. The efficiency of cross-linking reactions was monitored by immunoblotting analyses. Upon optimization of this procedure, cross-linked proteins of high molecular weight (higher than the bait proteins) were readily detected; these proteins were not detected in non-cross-linked controls, suggesting the effectiveness of this method (Fig. 1b). Comparative analyses of cross-linked samples and controls led to the identification of most known SopD2-interacting proteins (e.g., Rab7, Rab8 and Rab10 in the left panel of Fig. 1c), demonstrating the efficacy of this strategy. Importantly, in cross-linked SetA samples but not in the controls, we detected multiple Rab GTPases (i.e., Rab1, Rab5c and Rab7) (Fig. 1c, the right panel). Together with previous findings on the disruption of host vesicle trafficking, our cross-linking mass spectrometry analyses suggest that Rab GTPases are valid host cell target candidates for SetA. The identification of host targets arguably is the greatest challenge in the study of effector function, probably due to the low enzyme-substrate affinity. The success of identifying Rab small GTPases as potential targets for SetA by cross-linking suggests that this method can be generalized for the study of other effectors.

### Ectopic expression of SetA caused glucosylation of Rab1 in mammalian cells

**Fig. 2:**
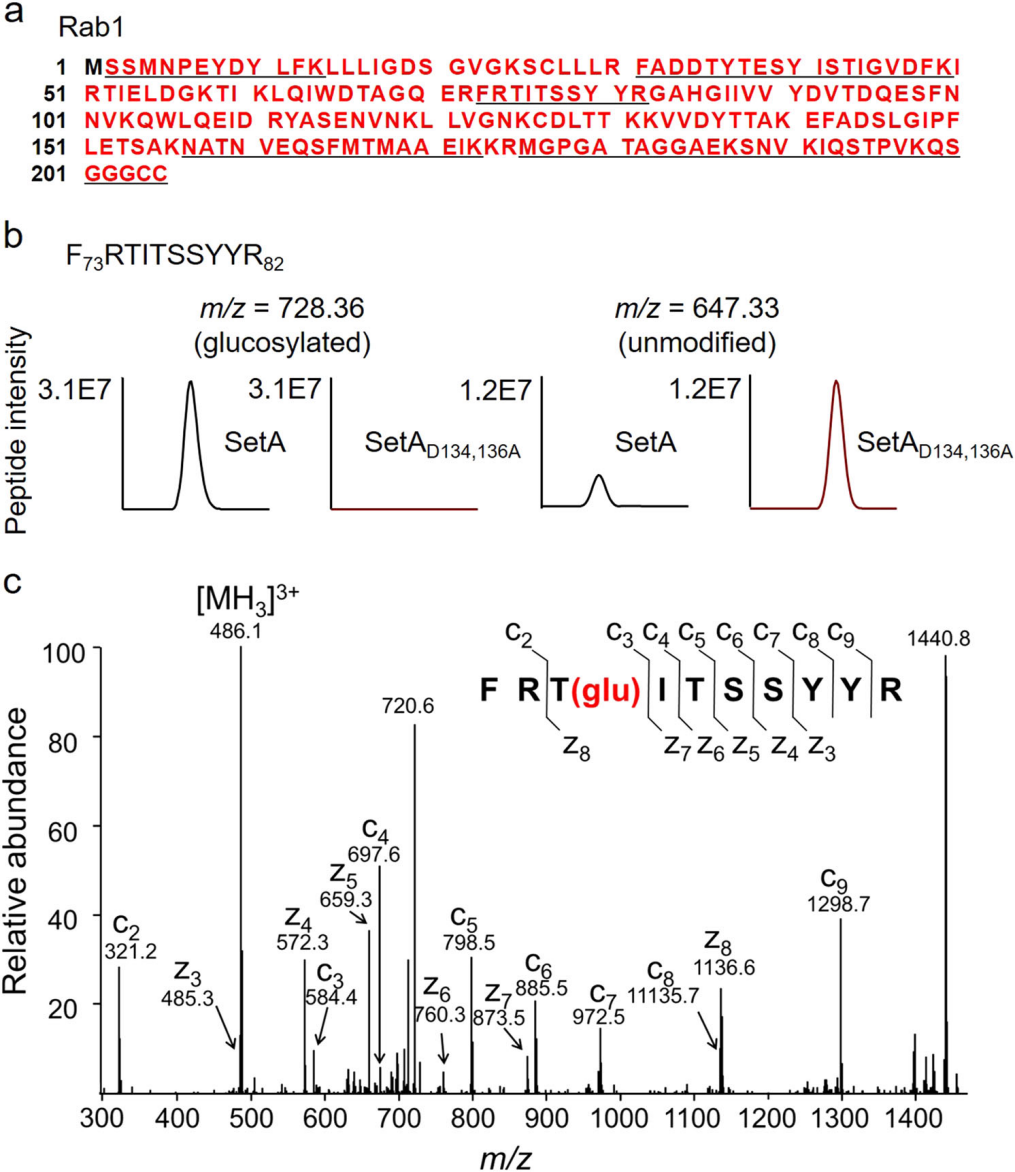
Extensive glucosylation of Rab1 upon co-expression with SetA in mammalian cells. 3×FLAG-Rab1 was isolated from HEK293T cells co-transfected with either wild-type SetA or its catalytically inactive mutant SetA_D134,136A_. Immunoprecipitated Rab1 was then digested with trypsin and analyzed by LC-MS/MS. **a** Detected Rab1 sequence shown in red in LC-MS experiments. The glucosylated peptide sequences are underlined. **b** MS detection of Rab1 peptide F_73_RTITSSYYR_82_ that was covalently modified with one molecule of glucose. Extracted ion chromatograms of the doubly protonated peptide are shown with peak intensities indicating the relative amounts of either the modified (*m/z* = 728.36) or unmodified (*m/z* = 647.33) peptides. **c** Determination of modification sites by electron transfer dissociation (ETD) analysis. The MS/MS spectrum of modified F_73_RTITSSYYR_82_ is shown. The fragment ions c3 to c9 have a mass increase of 162.1 corresponding to the addition of one glucose while z3 to z7 fragments lack such a mass shift, suggesting glucosylation of Thr_75_

Next, we examined whether the Rab GTPases identified above are glucosylation targets of SetA. We co-expressed 3×FLAG-tagged Rab1 in HEK293T cells with either wild-type (WT) SetA or its catalytically inactive mutant SetA_D134,136A_. With nearly full coverage of the Rab1 sequence, we detected seven mono-glucosylated peptides (Fig. 2a), suggestive of multiple modification sites catalyzed by SetA. By quantitative mass spectrometry, we determined the extent (i.e., percentages) of glucosylation for these modified peptides. Our data reveal that peptide -F_73_RTITSSYYR_82_- was highly (~75%) modified (Fig. 2b). Interestingly, this fragment is in the switch II region of Rab1 and contains the modification sites for both SidM and AnkX-dependent AMPylation and phosphorylcholination^14,17,18^. In contrast, the percentage of modification for most of the other modified peptides was below 5% (Supplementary Table S2). In addition, we examined Rab5c and Rab7 co-expressed with SetA and found only a small (<5%) fraction of the peptides was glucosylated (Supplementary Table S2, Supplementary Fig. S1 and S2). These findings suggest that among the identified small GTPases, Rab1 is likely to be the preferred substrate of SetA.

We thus focused our analysis on SetA-mediated glucosylation of Rab1. The doubly pronated peptide (*m*/*z* *=* 728.36) showed a mass shift of 162.05, corresponding to the attachment of one glucose molecule. In contrast, such an increase in mass was not observed in the peptide samples from Rab1 co-expressed with the enzymatically inactive mutant SetA_D134,136A_ (Fig. 2b). We then sought to pinpoint the exact site of modification within this peptide. Due to extensive neutral loss of sugar moieties in traditional MS/MS (i.e., collision-induced dissociation), we fragmented the modified peptide by electron transfer dissociation (ETD)^27^. MS/MS analysis unambiguously determined the glucosylated site at Thr_75_ (Fig. 2c). Taken together, these data suggest that production of SetA in mammalian cells caused mono-glucosylation of Rab1 at Thr_75_, a site in the vicinity of the modification sites (Ser_79_ and Tyr_80_) of AnkX and SidM, respectively^14,17,18^. As expected, SetA-mediated modification of Rab1 required its glucosyltransferase activity.

### Rab1 was glucosylated by purified SetA

**Fig. 3:**
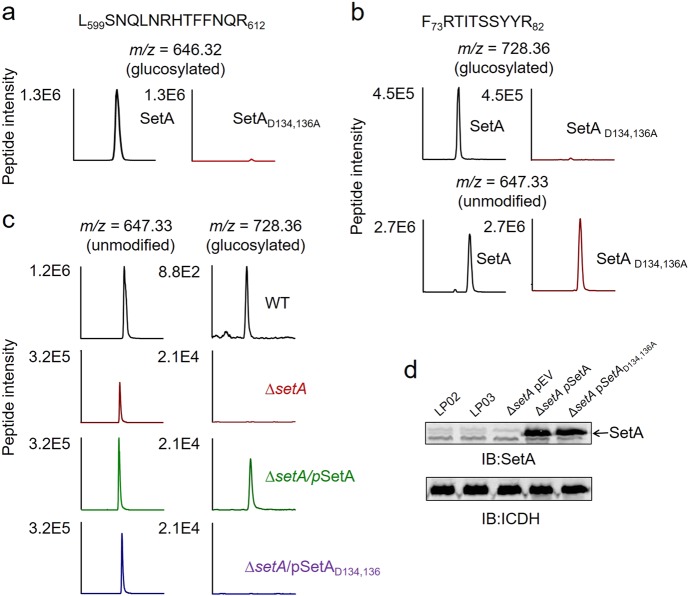
Glucosylation of Rab1 in biochemical reactions and during *L. pneumophila* infection. **a** Auto-glucosylation of SetA. Glucosylated SetA peptides were detected in wild-type SetA but not in its enzymatically inactive mutant. The extracted ion chromatograms of doubly protonated L_599_SNQLNRHTFFNQR_612_ (*m/z* = 646.32) are shown. **b** In vitro glucosylation assays with UDP-glucose as a precursor. Equal amounts of purified Rab1 were incubated with either His-SetA or its enzymatically inactive mutant SetA_D134,136A_. Gel-separated Rab1 was digested for further LC-MS/MS analyses. Extracted ion chromatograms of Rab1 peptide F_73_RTITSSYYR_82_ with glucosylation (*m/z* = 728.36) and without modification (*m/z* = 647.33) are shown. **c** Glucosylation of Rab1 by SetA during *L. pneumophila* infection. FLAG-tagged Rab1 was isolated from host cells infected by the indicated *L. pneumophila* strains and analyzed by LC-MS/MS. The extracted ion chromatograms of Rab1 peptide F_73_RTITSSYYR_82_ are shown. **d** The expression levels of SetA in different *L. pneumophila* strains. Lp02: wild type; Lp03: *dotA*;^-^ Δ*setA*pEV: the Lp02Δ*setA* strain carrying an empty vector; Δ*setA*pSetA: the Lp02Δ*setA* strain carrying a plasmid that expresses SetA; Δ*setA*pSetA_D134,136A_: the Lp02Δ*setA* strain carrying a plasmid that expresses the enzymatically inactive mutant SetA_D134,136A_

Next, we asked whether SetA was capable of directly modifying Rab1 by glucosylation. We first examined the glucosyltransferase activity of SetA by incubating purified recombinant His_6_-SetA or its catalytically inactive mutant His_6_-SetA_D134,136A_ with UDP-glucose. LC-MS readily detected the auto-glucosylation products of SetA. The glucosylated peptide -L_509_SNQLNRHTFFNQR_612_- (*m*/*z* *=* 646.32, *z* = 3) was present in the samples from wild-type SetA but not the catalytically inactive mutant (Fig. 3a). Then we performed glucosylation assays by incubating purified GST-Rab1 and UDP-glucose with either His_6_-SetA or His_6_-SetA_D134,136A_. The purity of these recombinant proteins was higher than 95% as determined by SDS-PAGE analysis (Supplementary Fig. S3). Glucosylated Rab1 peptide -F_73_RTITSSYYR_82_- was detected in reactions containing SetA but not the SetA_D134,136A_ mutant (Fig. 3b). In comparison to the co-expression experiments performed above, we observed a relatively lower efficiency of modification (~7%) probably due to less optimal reaction conditions used in these biochemical assays. To confirm the site of modification, we constructed point mutations (Rab1_T75A_ and Rab1_T77A_) and analyzed the glucosylation of these mutants in vitro. When threonine 75 was mutated to alanine, we did not detect any signal corresponding to the modified peptide _73_FRAITSSYYR_82_. In contrast, when threonine 77 was substituted by alanine, the peptide _73_FRTIASSYYR_82_ can still be modified by SetA (Supplementary Fig. S4). Taken together, these results establish that SetA is a glucosyltransferase that directly modifies Rab1 at threonine 75. As co-expression with SetA also caused modifications of Rab5c and Rab7 (albeit at much lower efficiencies), we further examined whether SetA directly glucosylates these two GTPases. We detected a small fraction (~2%) of glucosylated Rab7 but no modification of Rab5c (Supplementary Fig. S5).

### SetA specifically glucosylated Rab1 during *L. pneumophila* infection

To further validate Rab1 as the physiological substrate of SetA, glucosylation during *L. pneumophila* infection was examined. To monitor the modification status of Rab1 during bacterial infection, we infected HEK293T cells expressing 4×FLAG-Rab1 with relevant *L. pneumophila* strains. Signals of the glucosylated peptide -F_73_RTITSSYYR_82_- were detected in cells infected by wild type but not the Lp02∆*setA* mutant (Fig. 3c). Importantly, introduction of a plasmid expressing SetA into the strain restored its ability to modify Rab1 (Fig. 3c). In contrast, although expressed at similar levels (Fig. 3d), SetA_D134,136A_ was unable to complement the ability of strain Lp02∆*setA* to glucosylate Rab1 (Fig. 3c). Consistent with higher expression and secretion levels of SetA produced from a multi-copy plasmid (Fig. 3d and Supplementary Fig. S6), the ratio of Rab1 glucosylation in cells infected with the complementation strain was more than 10 times higher than that in wild-type infected cells (Fig. 3c). Taken together, these findings show that Rab1 is the target of SetA for glucosylation during *L. pneumophila* infection.

As our previous experiments had revealed that ectopic expression of SetA led to modifications of Rab5c and Rab7 in mammalian cells and that SetA directly glucosylated Rab7, we examined whether these two GTPases are modified by SetA during *L. pneumophila* infection. Signals from modified peptides were not detected even in cells infected with the strain that overexpressed SetA (Supplementary Fig. S7). Thus, Rab1 is the specific substrate of SetA during *L. pneumophila* infection.

### SetA preferentially modified the GDP-bound form of Rab1 and the modification affected its interactions with GDI1 but not SidM

**Fig. 4:**
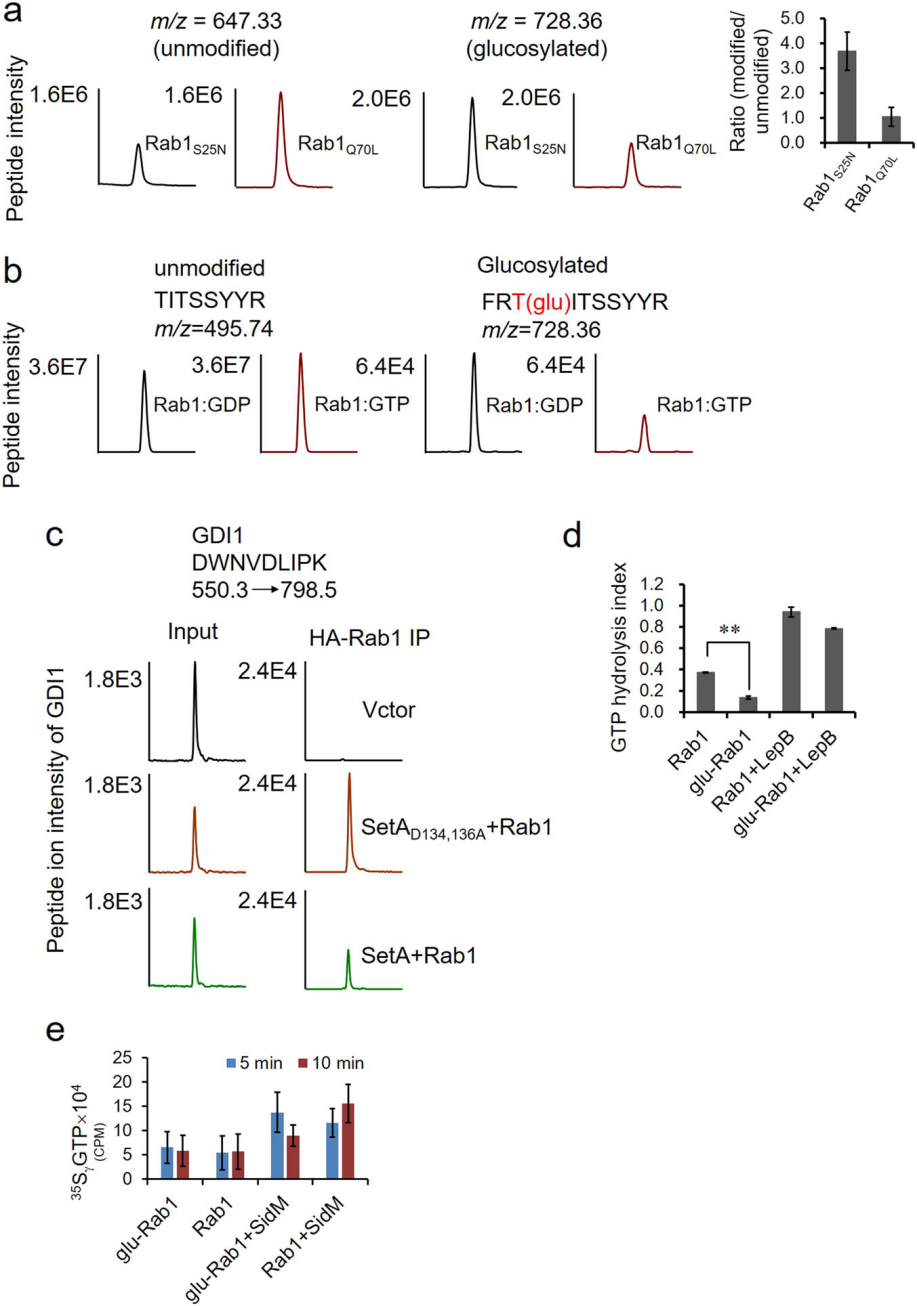
Preferential glucosylation of GDP-locked Rab1 over the GTP-bound form and the influence of this modification on Rab1 function. **a** SetA co-expressed with Rab1_Q70L_ (GTP-locked Rab1) or Rab1_S25N_ (GDP-locked Rab1) in HEK293T cells. The glucosylation of these two forms of Rab1 was detected by LC-MS/MS. The left panels are the extracted ion chromatograms of doubly protonated peptide F_73_RTITSSYYR_82_ with peak intensities representing the relative amounts of the modified or unmodified peptides. The right bar graphs plot the intensity ratio of the modified peptide over the unmodified one for both GTP- and GDP-locked Rab1 samples. **b** Wild-type Rab1 was purified and loaded with GDP or GTP. The in vitro glucosylation of Rab1:GDP or Rab1:GTP was monitored by LC-MS. The left and right panels are the extracted ion chromatograms of doubly protonated peptide T_75_ITSSYYR_82_ and F_73_RTITSSYYR_82_ with peak intensities representing the relative amounts of the unmodified and modified peptides respectively. **c** Glucosylation of Rab1 affected its interaction with GDI1. HA-tagged Rab1 was co-expressed with FLAG-SetA or its catalytically inactive mutant SetA_D134,136A_ in HEK293T cells. Samples from cells transfected with an empty vector were included as negative controls. The relative abundance of the endogenous GDI1 co-precipitated with Rab1 was quantified by selected reaction monitoring (SRM) analyses with the transition of 550.29 → 798.5 (DWNVDLIPK). **d** Inhibition of Rab1 GTPase activity by SetA-mediated glucosylation. 1 mM unmodified Rab1 or glucosylated Rab1 were incubated with GTP for 2 h with or without the addition of 0.1 mM LepB. The GTPase activity was assayed by measuring the level of free phosphate released by Rab1-mediated hydrolysis. The GTP hydrolysis index was calculated as follows: (OD_620_ of the experimental samples − OD_620_ of the blank)/OD_620_ of the Rab1 associated with LepB. **e** Impact of Rab1 GTP loading by SetA-mediated glucosylation. GDP-loaded GST-Rab1 or glucosylated GST-Rab1 was incubated with ^35^SγGTP with or without SidM for the indicated time for the GTP loading reaction. Radioactivity associated with the protein was determined by a scintillation counter. Data are from three independent experiments (**a**, **d**, **e**) with error bars denoting standard deviation. *p < 0.05, **p < 0.01

Rab1 oscillates between a GTP-bound and a GDP-bound form in its activity cycle^28^. To determine the effects of the modification, we examined whether SetA has a preference for Rab1 in one of these two forms. We first ectopically expressed SetA in HEK293T cells together with either Rab1_Q70L_, a mutant that mimics the GTP-bound form^29^ or Rab1_S25N_, a mutant that assumes the GDP-bound conformation^30^. Immunoblotting assays indicate that both SetA and Rab1 were produced at similar levels between cells transfected to express these two mutants (Supplementary Fig. S8). Each form of Rab1 was then affinity purified for analysis by LC-MS to determine the ratios of modification. Our results reveal that Rab1 in the GDP-bound form exhibited a markedly higher ratio of modification than that of its GTP-bound form (Fig. 4a). Furthermore, we evaluated the in vitro modification ratios by loading purified Rab1 with either GDP or a non-hydrolyzable GTP analog GTPγS. Upon incubation with SetA, LC-MS analyses revealed a higher modification ratio (2–3 fold) of Rab1:GDP than that of Rab1:GTP (Fig. 4b). Collectively, these results established that SetA preferentially modifies the GDP-bound form of Rab1.

In the regulation of vesicle trafficking, Rab proteins are cycled between the cytosol and intracellular membranes depending on their activation states^31^. In the inactive GDP-bound form, Rabs bind to a GDP dissociation inhibitor (GDI) and are trapped in the cytosol. In the active GTP-bound form, Rabs are associated with membranes, where they interact with effectors to promote vesicle fusion and trafficking^32^. As SetA preferentially modifies the GDP-bound form of Rab1 and AMPylation or phosphorylcholinationof Rab1 inhibits its binding to a GDI^33^, we wondered whether glucosylation of Rab1 had a similar effect. We expressed HA-Rab1 in mammalian cells together with either FLAG-SetA or FLAG-SetA_D134,136A_ and quantified the relative abundance of the endogenous GDI1 co-precipitated with Rab1 by selected reaction monitoring (SRM) analyses. Immunoprecipitation of the potential Rab1–GDI1 complex showed markedly less GDI1 binding to glucosylated Rab1 than the unmodified protein prepared from cells producing the SetA mutant (Fig. 4c).

Activation of Rab1 requires the exchange of GDP for GTP with the aid of a GEF protein. The Dot/Icm effector SidM is a GEF that directly binds to Rab1 and recruits it to the LCV^11–13,34^. We tested whether glucosylation of Rab1 affects its interaction with SidM. We thus compared the binding affinity of SidM to glucosylated Rab1 and its native form. Purified recombinant SidM was incubated with lysates from cells expressing Rab1 together with either SetA or the catalytically dead mutant. Immunoprecipitation of the potential Rab1–SidM complex showed indistinguishable binding of SidM to glucosylated Rab1 and unmodified controls prepared from cells producing the SetA mutant (Supplementary Fig. S9). Together, these results suggest that glucosylation of Rab1 affects its interaction with GDI1 but not SidM, which is similar to the impact of AMPylation or phosphorylcholination on this GTPase^14,17,33^.

### Glucosylation of Rab1 inhibited its GTPase activity but did not interfere with GTP loading

Next, we investigated the functional consequences of SetA-mediated glucosylation on its GTP hydrolysis activity. To obtain large amounts of modified proteins, GST-Rab1 was overexpressed in *E. coli* together with His_6_-SetA or His_6_-SetA_D134,136A_. LC-MS measurements of the affinity purified Rab1 showed that >70% of protein was glucosylated when co-expressed with SetA (Supplementary Fig. S10). Both the modified and unmodified versions of Rab1 were purified and incubated with GTP in reactions with or without the bacterial GAP LepB^11^. Compared to non-modified controls, glucosylated Rab1 exhibited markedly lower GTPase activity (Fig. 4d). As expected, in reactions that received LepB, the GTP hydrolysis activity was significantly higher. Nevertheless, the modified Rab1 exhibited significantly lower efficiency of GTP hydrolysis compared to its native counterpart (Fig. 4d).

To better understand the inhibition of Rab1 GTPase activity upon glucosylation, we examined the loading of GTP with or without SidM to the modified Rab1. Spontaneous GTP loading by glucosylated Rab1 did not detectably differ from its unmodified counterpart (Fig. 4e). Thus, glucosylation inhibits the GTPase activity of Rab1 but not its ability to associate with GTP.

### Some modifications on Rab1 interfere with further effector-induced modifications

**Fig. 5:**
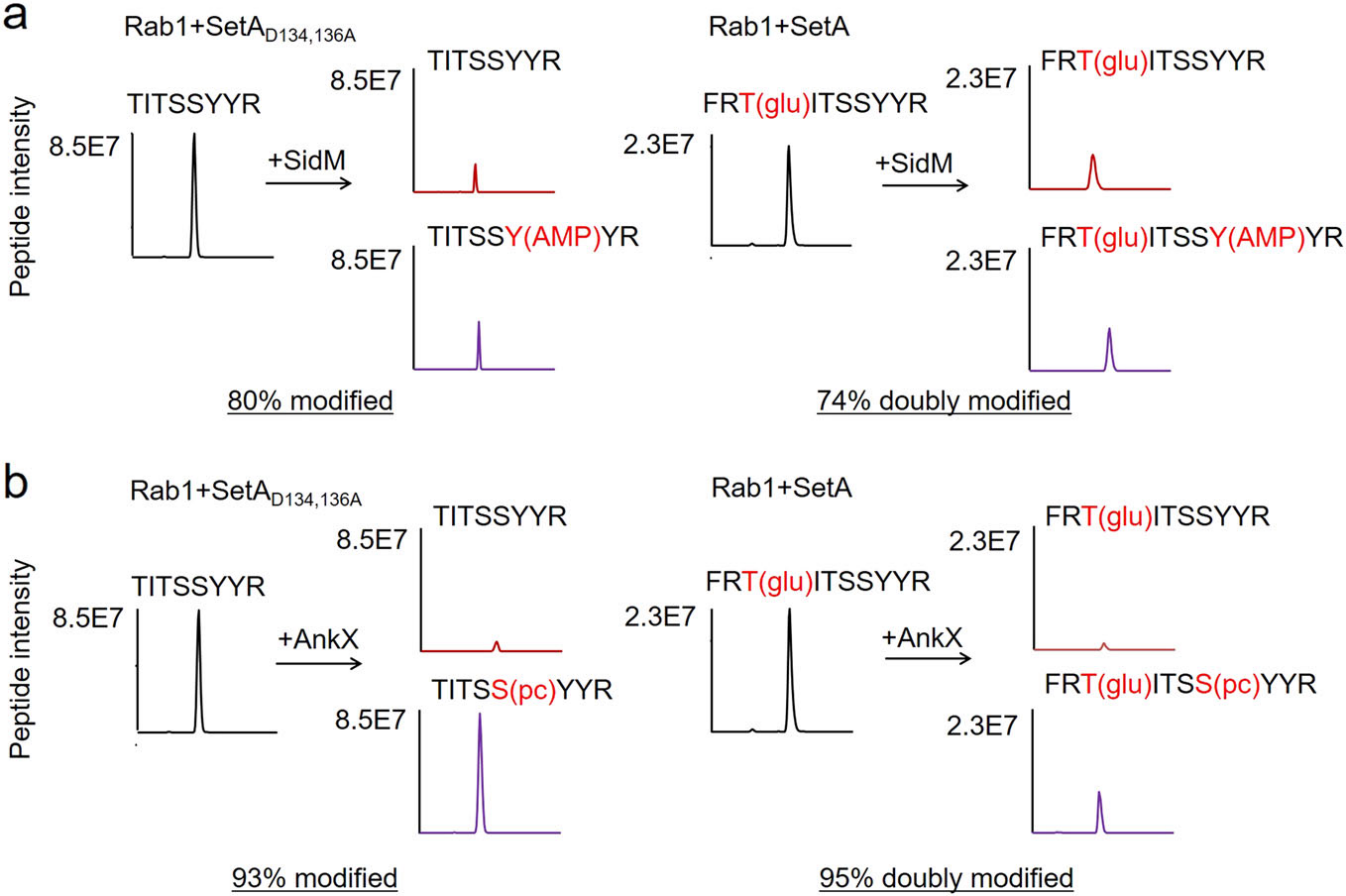
Primary glucosylation of Rab1 on Thr_75_ did not interfere with subsequent AMPylation or phosphorylcholination. Purified GST-Rab1 from *E. coli* expressing SetA or its catalytically inactive mutant was incubated with either SidM or AnkX for potential AMPylation or phosphorylcholination. The modification status of Rab1 was analyzed by LC-MS/MS. The extracted ion chromatograms of different peptides (with or without modifications) are shown. The ratios of AMPylation or phosphorylcholination of unmodified T_75_ITSSYYR_82_ and glucosylated F_73_RTITSSYYR_82_ were assessed by monitoring the peak intensities of various peptide forms before and after in vitro reactions. **a** The ratios of AMPylation of unmodified T_75_ITSSYYR_82_ and glucosylated F_73_RTITSSYYR_82_. **b** The ratios of phosphorylcholination of unmodified T_75_ITSSYYR_82_ and glucosylated F_73_RTITSSYYR_82_

The residue Thr_75_ glucosylated by SetA is close to the sites modified by AnkX and SidM (Ser_79_ and Tyr_80_, respectively)^14,17^. Next, we set out to determine whether primary glucosylation of Rab1 interferes with subsequent AMPylation or phosphorylcholination due to potential steric hindrance. To address this question, we purified GST-Rab1 from *E. coli* expressing either SetA or the catalytically inactive SetA_D134,136A_ and further incubated the proteins with either SidM or AnkX for potential secondary modifications (i.e., double modifications on the same protein). The images of SDS-PAGE gels with all proteins used in the reactions were shown (Supplementary Fig. S11). In all cases, the ratios of modifications were monitored by LC-MS measurements of relevant peptides. Double modifications of Rab1 (simultaneous glucosylation together with AMPylation or phosphorylcholination) were readily observed, as evidenced by the detection of the doubly modified peptides -F_73_RT(glu)ITSS(pc)YYR_82_- and -F_73_RT(glu)ITSSY(AMP)YR_82_- under collision-induced dissociation (Supplementary Fig. S12). Quantitative mass spectrometric analyses indicated that approximately 74% of glucosylated Rab1 was further AMPylated upon incubation with SidM, yielding dual modified proteins; this ratio did not differ significantly from reactions with unmodified Rab1 (~80%) (Fig. 5a). Similar results were obtained for glucosylated Rab1 used for subsequent phosphorylcholination (93% vs. 95%) (Fig. 5b).

Additionally, we investigated whether primary AMPylation or phosphorylcholination would impact subsequent glucosylation. Purified Rab1 was first incubated with either SidM or AnkX prior to the addition of SetA and UDP-glucose. LC-MS analyses detected markedly lower glucosylation ratios in AMPylated or phosphorylcholinated Rab1 (Supplementary Fig. S13). In fact, peptides modified by both phosphorylcholination and glucosylation were not detectable under our experimental conditions. Taken together, these findings suggest that primary glucosylation of Rab1 did not interfere with subsequent AMPylation or phosphorylcholination, whereas AMPylation or phosphorylcholination affected secondary glucosylation.

## Discussion

To establish an intracellular niche permissive for its replication, *L. pneumophila* utilizes a large number of effectors to hijack host vesicle trafficking pathways^5^. Among these, SetA inhibits yeast growth by targeting vesicle trafficking in a manner that requires a glucosyltransferase domain containing the conserved DXD motif, which possesses mono-*O*-glucosyltransferase activity by using UDP-glucose as a sugar donor^22,23^. Glycosylation is increasingly recognized as an important strategy used by bacterial pathogens to subvert host cell functions. *Clostridium difficile* toxins A (ToxA) and B (ToxB), for instance, glucosylate Rho GTPases, leading to a redistribution of the microfilament system^35,36^. In addition, the *E. coli* type III effector NleB catalyzes arginine GlcNAcylation of host death receptors to disrupt TNF signaling in infected cells^37,38^. Interestingly, *L. pneumophila* itself also encodes three other effectors (Lgt1, 2, and 3) with glucosyltransferase activity. Unlike SetA, these effectors function to inhibit host protein synthesis by attacking the elongation factor eEF1A^39,40^, probably to liberate amino acids for bacterial consumption^41^.

Rab GTPases cycle between an inactive GDP-bound form and an active GTP-bound form to recruit different downstream effectors responsible for vesicle formation, movement, tethering and fusion^31,42^. Mass spectrometry analyses revealed that multiple residues of Rab1 were glucosylated when SetA was overexpressed (Fig. 2a and Supplementary Table S2). Quantitative mass spectrometric analysis showed that modification on Thr_75_ had the highest ratio when Rab1 was co-expressed in mammalian cells (Fig. 2b and Supplementary Table S2). Second, in reactions with purified proteins, Rab1 was effectively modified by SetA, again mostly on Thr_75_ (Fig. 3b). Further, only the modification of this site was detectable in Rab1 purified from cells infected by *L. pneumophila* (Fig. 3c). Thus, Thr_75_ is the major site of modification. Interestingly, this residue locates in the highly conserved switch II region of GTPases, which is involved in binding to various regulatory proteins. Importantly, Levin et al. found that Thr_75_ is phosphorylated by TAK1, a kinase involved in innate immunity^43^. In addition, when infected by *L. pneumophila* mutant Δ*ankX*Δ*sidM*, the level of Rab1 phosphorylation was lower than those in uninfected cells or cells infected by the Δ*dotA* mutant^43^, suggesting additional effector(s) may target Rab1 and influence TAK1-mediated phosphorylation. We reason that SetA may be one of these effectors, which compete with TAK1 for available substrates. Clearly, the bacterium contends with the host to control the activity of Rab1. Phosphorylation on Thr_75_ reduces the binding affinity of Rab1 to GDI1^43^. Glucosylation on Thr_75_ also led to diminished interactions between Rab1 and GDI1 (Fig. 4c). One possible explanation is that glycosylation of Rab1 somewhat disrupts its interaction with REP (Rab escort protein), leading to decreased prenylation and hence binding to GDI1. From another perspective, decreased Rab1/GDI1 interaction would promote Rab1 incorporation into the membrane of the LCV, consistent with the finding that SetA is associated with the LCV shortly after *Legionella* uptake^23^. Nonetheless, the Δ*setA* mutant did not exhibit detectable difference in the retention of Rab1 on the LCV, arguing against a role of SetA-mediated glycosylation in altering the cellular localization of Rab1.

Our results indicate that glucosylation inhibits the GTPase activity of Rab1 but does not affect its ability to receive GTP (Fig. 4d, e), which is in line with the observation that SetA prefers the GDP-bound form of Rab1. Thus, the activity of SetA appears to increase the pool of the GTP-bound, active form of Rab1. In this regard, SetA may function synergistically with other effectors such as SidM to ensure that active Rab1 is associated with the LCV for a certain duration during *L. pneumophila* infection.

Of note is that despite the proximity of the major glucosylation site on Rab1 to residues attacked by *L. pneumophila* effectors SidM and AnkX, modification of Thr_75_ by SetA does not detectably interfere with subsequent AMPylation of Tyr_80_ or phosphorylcholinationof Ser_79_ (Fig. 5a, b). This observation suggests that steric hindrance is not an issue for simultaneous Rab1 modifications by multiple effectors. However, we found that secondary glucosylation was impaired by AMPylation or phosphorylcholination (Supplementary Fig. S13). We reason that the first modification may induce some conformational changes, rendering the substrate less accessible by SetA. Intriguingly, we did not detect MS signals corresponding to any doubly modified Rab1 purified from cells infected with wild type *L. pneumophila*, suggesting that these modifications are not extensive enough for detection or simultaneous modifications may not occur on the same molecule during infection.

Our findings that SetA targets Rab1 by glucosylation provide a molecular mechanism for its blockage of the host secretory pathways as well as cellular toxicity to yeast and mammalian cells, effects which are also seen for SidM and AnkX^16,18^. The toxicity likely results from the lock of Rab1 in its active GTP-bound form, or from the disruption of its interactions with other cellular binding partners or a combination of both. The activity of SetA adds an additional layer of complexity to the regulation of Rab1 function. It is possible that host cells also regulate Rab1 activity by glucosylation at Thr_75_. For the study of *L. pneumophila* virulence, a future challenge is to dissect the potential interplays among these modifications and how each of them is temporally and spatially regulated to ensure a successful infection.

## Materials and methods

### Bacterial strains and plasmids

Bacterial strains and plasmids used in this study are listed in Supplementary Table S1. All *L. pneumophila* strains were derivatives of the Philadelphia 1 strain Lp02^44^. *E. coli* strains were grown and maintained on LB agar with the addition of antibiotics when necessary. Strains of *L. pneumophila* were grown and maintained on CYE agar or in AYE broth as previously described^44^. The Lp02∆*setA* strain was constructed as previously described^45^. Briefly, the flanking regions on either side of setA were amplified using the primer sets setAKO-up-F-SalI/setAKO-up-R-BamHI (ATTGTCGACAGTGCCGATCATGACGTTATTATAA/ ATTGGATCCTTGAGCCTCTTGACCAGCCTGTGGT) and setAKO-down-F-BamHI/setAKO-down-R-SacI (ATTGGATCCTCAAAGGCAACCAGAAACCGGGCAA/ ATTGAGCTCGCACCACAAAAAATCGCCAAAAAAT). The DNA fragments were then inserted into the R6K vector pSR47s^46^ using three-way ligation. The construct was introduced to strain Lp02 using tri-parental mating and clones carrying the vector backbone containing the flanking region inserts were selected for using CYE with kanamycin and streptomycin^45^. The clones were then passaged on CYE with 5% sucrose to select for bacterial cells that no longer carried the vector backbone. Finally, mutants carrying the deletion were identified by PCR. For complementation experiments, SetA or SetA_D134,136A_ was expressed from the RSF1010-derived plasmid pZL507^47^. Antibiotics were added as required with the following final concentrations: streptomycin, 30 μg/mL (100 μg/mL for *Legionella*); ampicillin, 50 μg/mL; kanamycin, 50 μg/mL (20 μg/mL for *Legionella*).

### Cell culturing and transfection

HEK293T cells were cultured in Dulbecco’s Modified Eagle Medium (DMEM, Hyclone) supplemented with 10% (v/v) fetal bovine serum (FBS, Gibco, Life Technologies) under an atmosphere of 5% CO_2_ at 37 °C. For transfection, HEK293T cells were seeded at a density of 6 × 10^5^ cells per 10 cm dish and cultured for 24 h. For cross-linking and immunoprecipitation experiments, 15 μg of plasmids expressing HA- and FLAG-tagged SetA or SopD2 were transfected into cells of 80% confluence by using the transfection reagent VigoFect (Vigorous). After 24 h cultivation, cells were lysed for in vivo formaldehyde cross-linking reactions and further immunoprecipitation. To examine whether the identified Rab GTPases are glucosylation targets of SetA, 10 μg of plasmids expressing HA- and FLAG-tagged SetA or SetA_D134,136A_ were co-transfected with 5 μg of plasmids expressing HA- and FLAG-tagged Rab1, Rab5c or Rab7 respectively. The Rab GTPases were further immunoprecipitated for the glucosylation assays. To examine whether SetA has a preference for a GTP-bound or GDP-bound form of Rab1, 10 μg of plasmids expressing HA- and FLAG-tagged SetA were co-transfected with 5 μg of plasmids expressing HA- and FLAG-tagged Rab1_Q70L_ or Rab1_S25N_ respectively. Each form of Rab1 was then affinity purified for LC-MS analyses to determine the ratios of modification. For analyzing the binding ability of unmodified or modified Rab1 to GDI1 or SidM, 10 μg of plasmids expressing FLAG-tagged SetA or SetA_D134,136A_ were co-transfected with 5 μg of plasmids expressing HA-tagged Rab1. GDI1 or SidM bound to Rab1 were co-precipitated and analyzed by LC-MS.

### In vivo formaldehyde cross-linking

HEK293T cells expressing HA- and FLAG-tagged SetA or SopD2 were trypsinized and pelleted in 1.5 mL reaction tubes. The pellets were washed once in PBS and resuspended in 1 mL of PBS. In vivo formaldehyde cross-linking of intact cells was carried out in PBS buffer by adding 27 μL of 37% formaldehyde at 37 °C for 10 min. The cross-linking reaction was quenched for 10 min at 30 °C by the addition of 0.125 M glycine. After cross-linking, cells were pelleted and washed once with PBS. Then cells were lysed for further immunoprecipitation and LC-MS analyses.

### Immunoprecipitation

For immunoprecipitation, cells expressing bait proteins were lysed in 1 mL of lysis buffer containing 150 mM Tris-HCl (pH 7.5), 150 mM NaCl, and 1% Triton. The lysates were clarified at 12,000 × *g* for 15 min to remove cell debris and the supernatants were incubated with anti-HA or anti-FLAG agarose beads (Sigma-Aldrich) overnight at 4 °C. For cross-linking immunoprecipitation, we used anti-HA agarose beads to minimize the adverse impact of cross-linking on the affinity between antibodies and bait proteins. The beads with bound proteins were washed four times with 1 mL of lysis buffer. Finally, the bound proteins were eluted by FLAG or HA peptides and boiled for 5 min in the SDS-PAGE sample buffer containing 60 mM Tris-HCl (pH 6.8), 1.7% (w/v) SDS, 6% (v/v) glycerol, 100 mM dithiothreitol (DTT), and 0.002% (w/v) bromophenol blue. Then the eluted samples were stored at −20 °C prior to further analyses.

### Bacterial infection

HEK293T cells were transfected with plasmids containing the gene for the FCγII receptor or the gene 4×FLAG-Rab1 using Lipofectamine 3000 (Life Technology) according to the manufacturer’s instructions. After 24 h, the cells were infected with *L. pneumophila* strains Lp02, Lp02∆*setA*, Lp02∆*setA*(pSetA) and Lp02∆*setA*(pSetA_D134,136A_) opsonized with rabbit anti-*Legionella* antibodies at 1:500 for 1 h at an MOI of 100. The infection was allowed to proceed for 30 min, after which the cells were collected, lysed in RIPA buffer (Thermo Fisher Scientific) and the 4×FLAG-Rab1 was immunoprecipitated using FLAG beads (Sigma-Aldrich). The M2 beads were then washed three times with RIPA buffer and three times with TBS (20 mM Tris-HCl pH = 8.0, 150 mM NaCl). The 4 × FLAG-Rab1 was competitively eluted from the FLAG beads using 3 × FLAG peptide at a concentration of 500 μg/mL. The eluted protein was concentrated, treated with SDS-PAGE sample buffer, boiled for 10 min and separated by SDS-PAGE. Samples (Coomassie stained gel slices) were further processed for LC-MS analysis.

### Immunoblotting analysis and antibodies

Rabbit polyclonal serum against SetA was produced by Jiaxuan Biotech Company (Shanghai, China). Antibody-containing serum was further affinity-purified against SetA covalently coupled to an Affigel matrix (Bio-Rad) using standard protocols^48^. For immunoblotting, the protein samples were separated by SDS-PAGE and transferred onto polyvinylidene difluoride (PVDF) membranes. After blocking with 5% milk for 1 h, membranes were incubated with the appropriate primary antibodies: anti-SetA (Jiaxuan Biotech, China, 1:200,000), anti-FLAG (Cwbio, China, 1:2500), anti-HA (Cwbio, China, 1:2500), anti-His (Cwbio, China, 1:2500), anti-GDI1 (abcom, China, 1:2500), anti-ICDH (Serum specific for Bacillus subtilis ICDH was generously provided by A. L. Sonenshein, Tufts University Medical School, Boston, MA and was used at 1:10,000) overnight at 4 °C. Then the membranes were washed 4 times with Tris-buffered saline containing 0.1% (v/v) Tween 20 (TBST), and incubated with horseradish peroxidase (HRP)-conjugated secondary antibodies (Cwbio, China, 1:5000) for 2 h at room temperature. After washing four times with TBST, antibody bands were visualized with the enhanced chemiluminescent (ECL) reagents (Tanon, China) by using a Tanon-5200 Image System (Tanon, China).

### Protein purification

The *E. coli* strain BL21(DE3) was used as the host for expression and purification of recombinant proteins. Rab1, Rab1_S25N_ and Rab1_Q70L_ were purified as GST-fusion proteins; SetA, SetA_D134,136A_, AnkX, SidM and LepB were purified as His_6_-fusion proteins. For protein purification, 10 mL of the overnight culture of the *E. coli* strain harboring the appropriate plasmids was transferred to 500 mL of fresh LB medium and grown at 37 °C until the OD_600_ value reached 0.6–0.8. The bacterial culture was allowed to cool down to 16 °C before the addition of isopropyl β-d-1-thiogalactopyranoside (IPTG) at a final concentration of 0.2 mM to induce protein expression. After overnight incubation (16–18 h) at 16 °C, bacterial cells were harvested by spinning at 5000 × *g* for 10 min and the pellets were resuspended in 30 mL of Tris-HCl buffer (25 mM, pH 7.5) containing 150 mM NaCl. Then bacterial cells were lysed by sonication on ice for 30 min. The lysates were centrifuged at 12,000 × *g* for 15 min to remove cellular debris and the supernatants were incubated for 2 h with either Ni-NTA or glutathione resins (GenScript) at 4 °C with gentle rotation. The protein-bound beads were washed three times with Tris-HCl buffer (25 mM, pH 7.5) containing 150 mM NaCl. Elution was carried out with 300 mM imidazole for His-tagged proteins and 25 mM reduced glutathione for GST fusion proteins. To produce guanine nucleotide-free Rab1 for GTP-loading and GTPase activity assays, GST-Rab1 was washed with PBS containing 20 mM EDTA before elution with 25 mM reduced glutathione. Eluted proteins were further dialyzed twice in a buffer containing 25 mM Tris-HCl (pH 7.5), 150 mM NaCl, 5% (vol/vol) glycerol, and 1 mM dithiothreitol (DTT).

### Preparation of GST-Rab1 of different activity status

The active form GST-Rab1:GTP were obtained using the nucleotide exchange method^49^. Briefly, 20 μL of GST-Rab1 attached to glutathione beads were washed with 100 μL of nucleotide exchange buffer (NE buffer: 20 mM HEPES, 100 mM NaCl, 10 mM EDTA, 5 mM MgCl_2_, 1 mM DTT, pH 7.5) containing 10 μM non-hydrolyzable GTP analog GTPγS and incubated for 10 min at room temperature in a 0.5 mL tube. The sample was centrifuged and the NE buffer was removed. Then 100 μL of NE buffer containing 1 mM GTPγS were added and incubated for 30 min under rotation. Subsequently, the NE buffer was removed again and the above procedures were repeated twice. Then the beads were washed with 100 μL of nucleotide stabilization buffer (NS buffer: 20 mM HEPES, 100 mM NaCl, 5 mM MgCl_2_, 1 mM DTT, pH 7.5) containing 10 μM GTPγS and further incubated with 100 μL of NS buffer in the presence of 1 mM GTPγS for 20 min at room temperature under rotation. For consistency, the GST-Rab1:GDP was obtained exactly as above except that the NE and NS buffers contained the same concentration of GDP instead of GTPγS. Finally, 10 μL of beads bound with GST-Rab1:GTP or GST-Rab1:GDP were used for in vitro glucosylation reactions.

### In vitro glucosylation reactions

1.4 μM of recombinant His_6_-SetA or His_6_-SetA_D134,136A_ was incubated for 1 h at 37 °C with 1 μM of GST-Rab1 in 20 μL of the reaction buffer containing 50 μM UDP-glucose, 1 mM MnCl_2,_ 20 mM Tris-HCl (pH 7.5) and 150 mM NaCl. For the analysis of substrate preference between two nucleotide-binding states, 1.4 μM of recombinant His_6_-SetA was incubated with 1 μM of GST-Rab1_S25N_ or GST-Rab1_Q70L_ under the same reaction conditions. Glucosylation reactions were terminated by boiling at 95 °C for 5 min in SDS-PAGE sample buffer. The reaction mixtures were separated by 10% SDS-PAGE and the corresponding Rab1 bands were processed for LC-MS/MS analysis.

### GTPase activity assay

GTPase activity was assayed by measuring the liberated phosphate from GTP hydrolysis using the malachite green method^47^. Briefly, 1 mM purified Rab1 (either glucosylated or unmodified) from SetA- or SetA_D134,136A_-expressing *E. coli* cells was incubated for 2 h at room temperature with 50 μL of GTPase reaction buffer (1 mM GTP, 10 mM HEPES, 125 mM KCl_,_ 5 mM MgCl_2_, pH = 7.0) with or without the addition of 0.1 mM LepB. Then 200 μL of the malachite green reagent (2 volumes of 0.0812% malachite green, 1 volume of 5.72% ammonium molybdate dissolved in 6 M HCl, 1 volume of 2.32% polyvinyl alcohol and 2 volumes of distilled water) was added. The reactions were allowed to proceed for 2 min and were terminated by the addition of 25 μL of 34% sodium citrate. After 30 min incubation, the absorbance at 620 nm was measured. A control with no enzymes was used as a blank.

### GTP-loading assay

GST-Rab1 was overexpressed in *E. coli* together with His_6_-SetA or His_6_-SetA_D134,136A_. Themodification ratios of affinity purified Rab1 were analyzed via mass spectrometry before testing the ability of each to load ^35^SγGTP (a non-hydrolyzable GTP analog). Nucleotide-free modified and unmodified GST-Rab1 (6.6 µM) were incubated in 100 μL nucleotide exchange buffer containing 25 mM Tris-HCl (pH 7.5), 50 mM NaCl, 5 mM MgCl_2_, and 0.1 mM EDTA with 5 mM unlabeled GDP for 2 h at room temperature. 15 μCi ^35^SγGTP (Perkin-Elmer) in 50 μL nucleotide exchange buffer was added to the samples. His_6_-SidM (5 μg) was added to indicated reactions to catalyze the loading of radiolabeled GTP analog. Reaction aliquots were withdrawn at indicated time points, placed onto nitrocellulose membrane filters (VSWP02500; Millipore) atop a vacuum platform attached to a waste liquid container. Membranes were washed three times using nucleotide exchange buffer to remove the free nucleotides, and were then transferred into scintillation vials containing 8 mL scintillation fluid (Beckman). Incorporated 35SγGTP was measured by a scintillation counter at 1 min per count.

### In-gel digestion and LC-MS/MS analysis

Upon SDS-PAGE fractionation, the band of interest was excised and subjected to in-gel trypsin digestion as previously described^50^. LC-MS analyses of protein digests were carried out on a hybrid ion trap-Orbitrap mass spectrometer (LTQ Orbitrap Velos, Thermo Scientific) coupled with nanoflow reversed-phase liquid chromatography (EASY-nLC 1000, Thermo Scientific). The capillary column (75 μm × 150 mm) with a laser-pulled electrospray tip (Model P-2000, Sutter instruments) was home-packed with 4 μm, 100 Å Magic C18AQ silica-based particles (Michrom BioResources Inc., Auburn, CA) and run at 250 nL/min with the following mobile phases (A: 97% water, 3% acetonitrile, and 0.1% formic acid; B: 90% acetonitrile, 10% water, and 0.1% formic acid). The LC gradient started at 7% B for 3 min and then was linearly increased to 37% in 40 min. Next, the gradient was quickly ramped to 90% in 2 min and stayed there for 10 min. The gradient was then switched back to 100% solvent A for column equilibration. Eluted peptides from the capillary column were electrosprayed directly onto the mass spectrometer for MS and MS/MS analyses in a data-dependent acquisition mode. One full MS scan (*m*/*z* 350–1500) was acquired by the Orbitrap mass analyzer with *R* = 60,000 and simultaneously the ten most intense ions were selected for fragmentation under collision-induced dissociation (CID) or electron transfer dissociation (ETD). Dynamic exclusion was set with repeat duration of 30 s and exclusion duration of 12 s.

### Data availability

The MS raw data have been deposited to the iProx database (URL:http://www.iprox.org/page/HMV006.html) and are available under the accession number IPX0001130001.

## Electronic supplementary material

Supplementary Information

## Electronic supplementary material

**Supplementary Information** accompanies the paper at (10.1038/s41421-018-0055-9).
