# Supplementary material for: Regulation of the small GTPase Rab1 function by a bacterial glucosyltransferase
Source: Cell Discov. 2018 Oct 9;4:53. doi: 10.1038/s41421-018-0055-9 (PMC6175885; doi:10.1038/s41421-018-0055-9)
Supplement: Supplementary file 1 — Supplementary Information [file 41421_2018_55_MOESM1_ESM.pdf]

Supporting Information

Table S1 Bacterial strains and plasmids used in this work.

**Strains**	**Description**	**References or sources**
L. pneumophila		
Lp02	Wild type	[49]
Lp03	*dotA*$^-$	[49]
Lp02*ΔsetA*		This study
Lp02*ΔsetA*(pSetA)		This study
Lp02*ΔsetA*(pSetA$_{134,136A}$)		This study
E. coli		
DH5α		TransGene Biotech
BL21-(DE3)		Gift from Youliang Yuan
**Plasmids**	**Properties**	**References or expressed proteins**
pET28a	For the expression of His$_6$-tagged proteins	SetA, SetA$_{D134,136A}$ SidM, AnkX, LepB
pcDNA4	Mammalian expression vector with N-terminal FLAG, HA, FLAG and HA tags	SetA, SetA$_{D134,136A}$, Rab1$_{S25N}$, Rab1$_{Q70L}$ Rab1, Rab5c, Rab7, SopD2
pGEX-6p1	*E. coli* GST fusion vector	Rab1, Rab1$_{S25N}$, Rab1$_{Q70L}$
pZL507	Vector for protein expression in *L. pneumophila*	[50]
pSetA	SetA cloned into pZL507	This study
pSetA$_{134,136A}$	SetA$_{134,136A}$ cloned into pZL507	This study
pSR47s	R6K vector for gene deletion	[50]

Table S2. The modification ratios of the glucosylated peptides from Rab1, Rab5c and Rab7.

**Protein**	**Peptide sequence**	**Peptide intensity**			**%Modification**[3]
		**Modified**	**Unmodified**[1]	**Unmodified**[2]	
**Rab1**	(73-82)FRTITSSYYR	3.7E8	5.3E7	2.1E8	75.0
	(31-49)FADDTYTESYISTIGVDFK	6.7E6	3.5E6	5.6E6	38.0
	(2-13)SSMNPEYDYLF	7.7E5	4.8E6	6.1E6	5.0
	(188-205)SNVKIQSTPVKQSGGGCC	5.1E6	4.8E7	3.5E7	5.0
	(188-198)SNVKIQSTPVK	5.3E6	5.5E7	4.9E7	3.0
	(176-187)MGPGATAGGAEK	1.2E7	3.3E8	4.6E8	1.0
	(157-173)NATNVEQSFMTMAAEIK	1.5E5	5.4E7	8.6E7	0.1
**Rab5c**	(83-92)YHSLAPMYYR	7.0E5	2.3E8	2.3E8	0.1
**Rab7**	(11-31)VIILGDSGVGKTSLMNQYVNK	1.2E5	2.4E6	3.4E6	2.0
	(22-31)TSLMNQYVNK	3.9E5	3.0E7	2.5E7	0.4
	(39-48)ATIGADFLTK	6.4E5	1.6E8	1.1E8	0.1
	(158-171)EAINVEQAFQTIAR	3.1E5	1.4E8	1.2E8	0.1

[1]: Intensity of unmodified peptides in the Rab samples prepared from cells expressing WT SetA.

[2]: Intensity of unmodified peptides in the Rab samples prepared from cells expressing enzymatically inactive SetA mutant.

[3]: Percentages are calculated as the ratio of the intensity drop of unmodified peptides in samples prepared from cells expressing WT SetA. When the modifications are minimal, the intensity of modified peptides was normalized first and then divided by that of unmodified peptides in the Rab samples prepared from cells expressing enzymatically inactive SetA mutant.

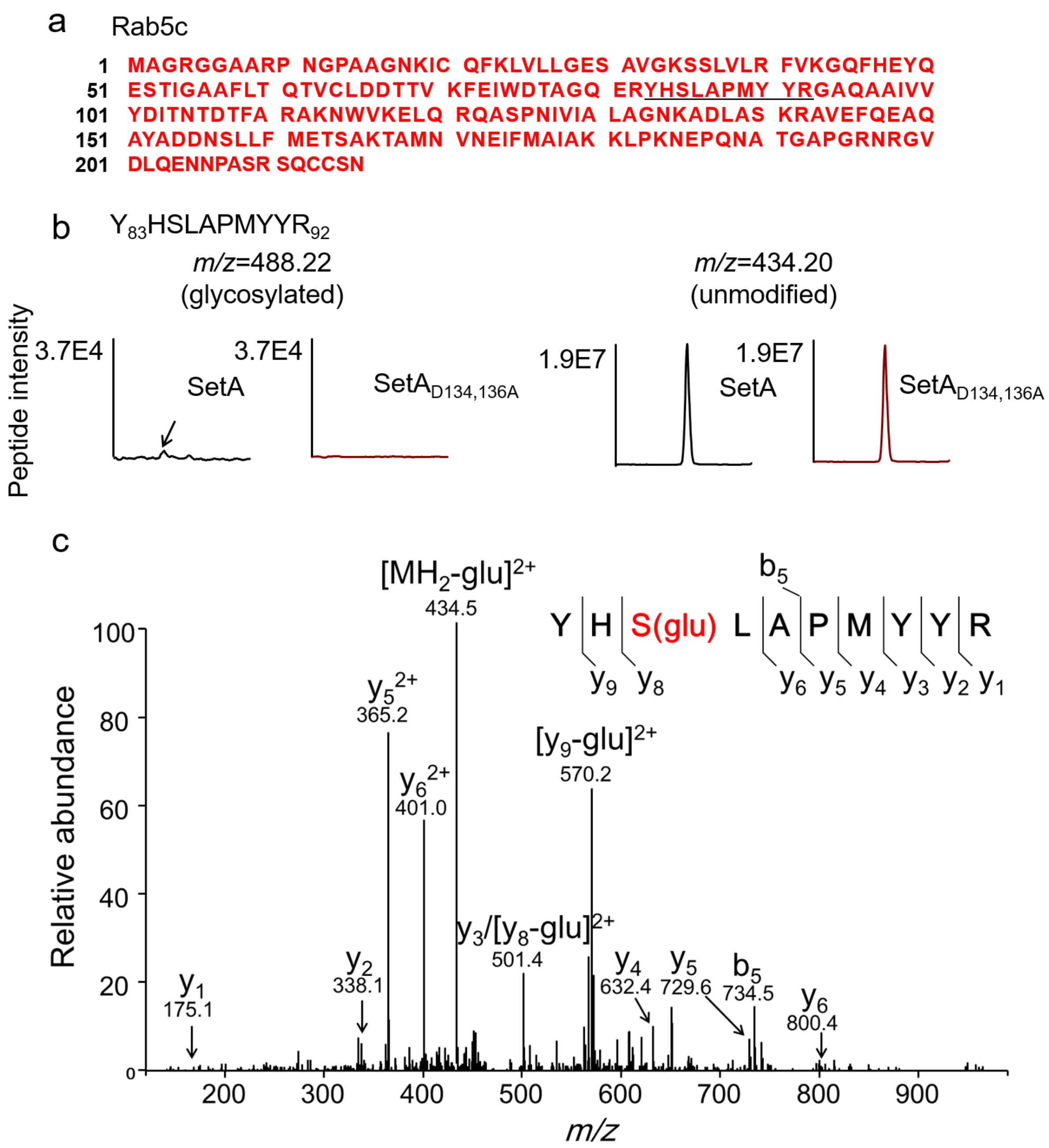

Figure S1. Glucosylation of Rab5c in SetA-expressing mammalian cells. (a) Detected Rab5c protein sequence (shown in red) by LC-MS/MS. 3×FLAG-Rab5c was immunoprecipitated from HEK293T cells co-transfected with SetA. The glucosylated peptide sequence is underlined. (b) MS detection of Rab5c peptide Y_{83}HSLAPMYYR_{92} and its glucosylated form. 3×FLAG-Rab5c was isolated from HEK293T cells co-transfected with either wild-type SetA or its catalytically inactive mutant $SetA_{D134,136A}$. The extracted ion chromatograms of doubly protonated Y_{83}HSLAPMYYR_{92} are shown. The peak intensities indicate the relative amounts of either modified YHSLAPMYYR (*m/z* = 488.22) or its unmodified version (*m/z* = 434.20). (c) The MS/MS spectrum of modified Y_{83}HSLAPMYYR_{92} acquired under collision-induced dissociation (CID).

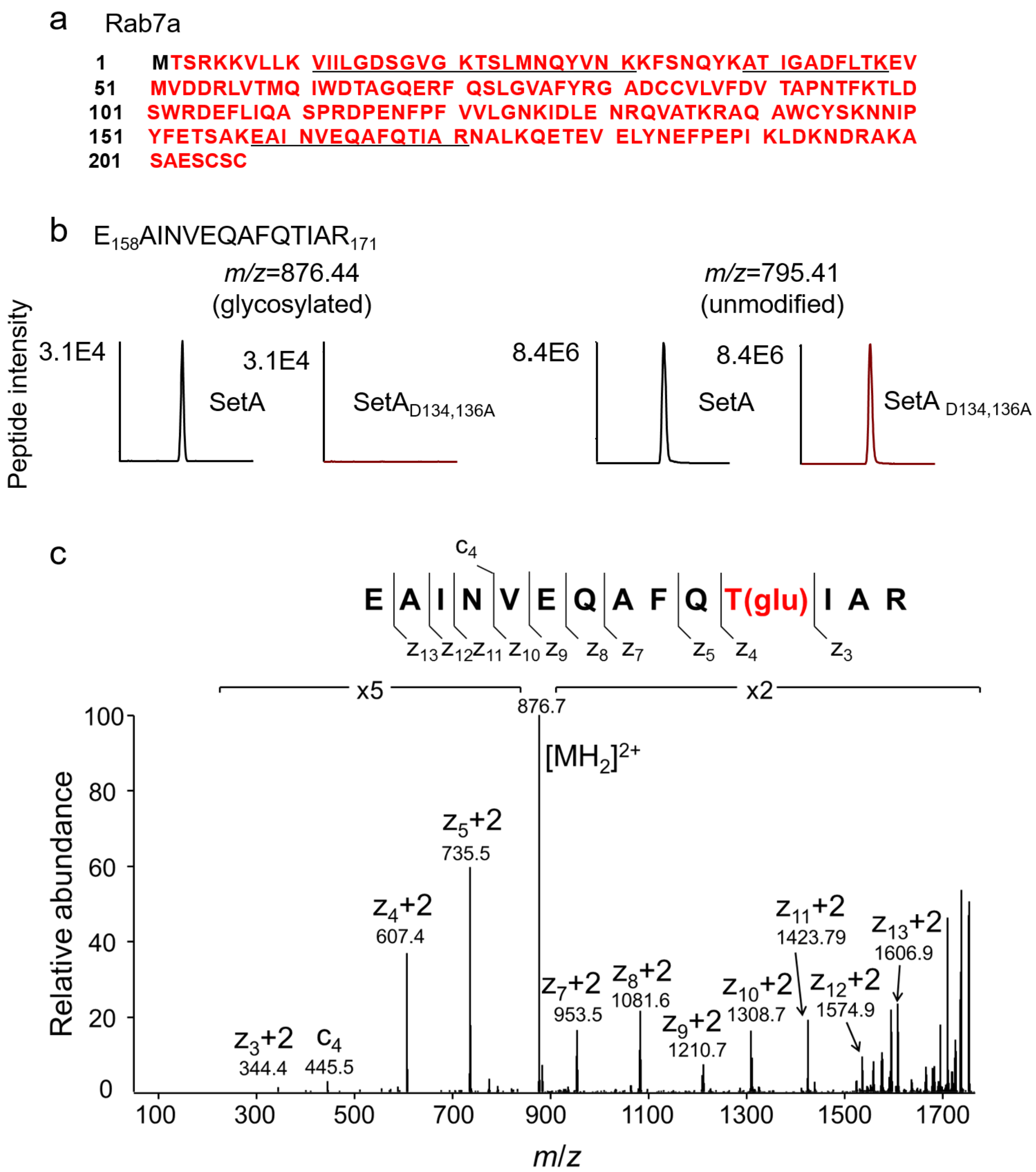

Figure S2. Glucosylation of Rab7 in SetA-expressing mammalian cells. (a) Detected Rab7 protein sequence (shown in red) by LC-MS/MS. 3×FLAG-Rab7 was immunoprecipitated from HEK293T cells co-transfected with SetA. The glucosylated peptide sequences are underlined. (b) MS detection of Rab7 peptide $E_{158}AINVEQAFQTIAR_{171}$ and its glucosylated form. 3×FLAG-Rab7 was isolated from HEK293T cells co-transfected with either wild-type SetA or its catalytically inactive mutant $SetA_{D134,136A}$. The extracted ion chromatograms of doubly protonated $E_{158}AINVEQAFQTIAR_{171}$ are shown. The peak intensities indicate the relative amounts of either modified $E_{158}AINVEQAFQTIAR_{171}$ (m/z = 876.44) or its unmodified version (m/z = 795.41). (c) The MS/MS spectrum of modified $E_{158}AINVEQAFQTIAR_{171}$ acquired under electron capture dissociation (ETD).

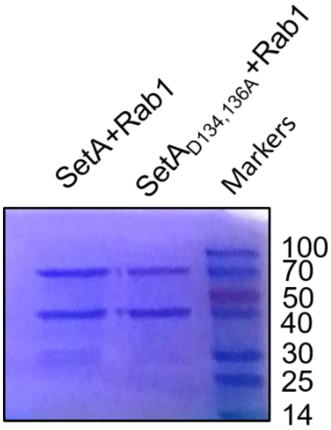

Figure S3. SDS-PAGE analyses of purified proteins used in biochemical reactions in Figure 3b.

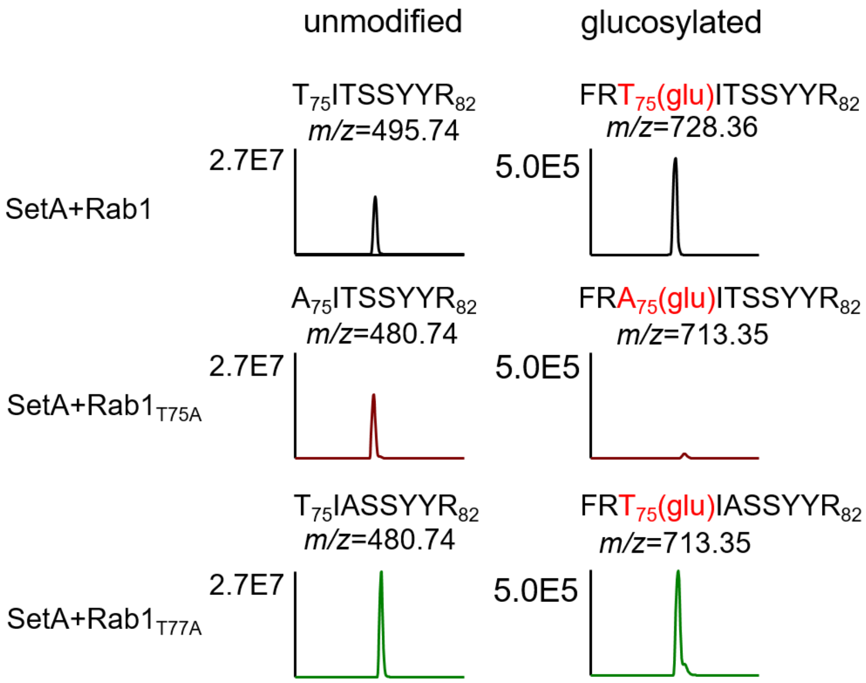

Figure S4. *In vitro* glucosylation assays of Rab1, Rab1$_{T75A}$ and Rab1$_{T77A}$. Equal amounts of purified Rab1, Rab1$_{T75A}$ or Rab1$_{T77A}$ were incubated with His-SetA. Gel-separated Rab1, Rab1$_{T75A}$ or Rab1$_{T77A}$ was digested for further LC-MS/MS analyses. The left and right panels are the extracted ion chromatograms with peak intensities representing the relative amounts of the unmodified and modified peptides respectively.

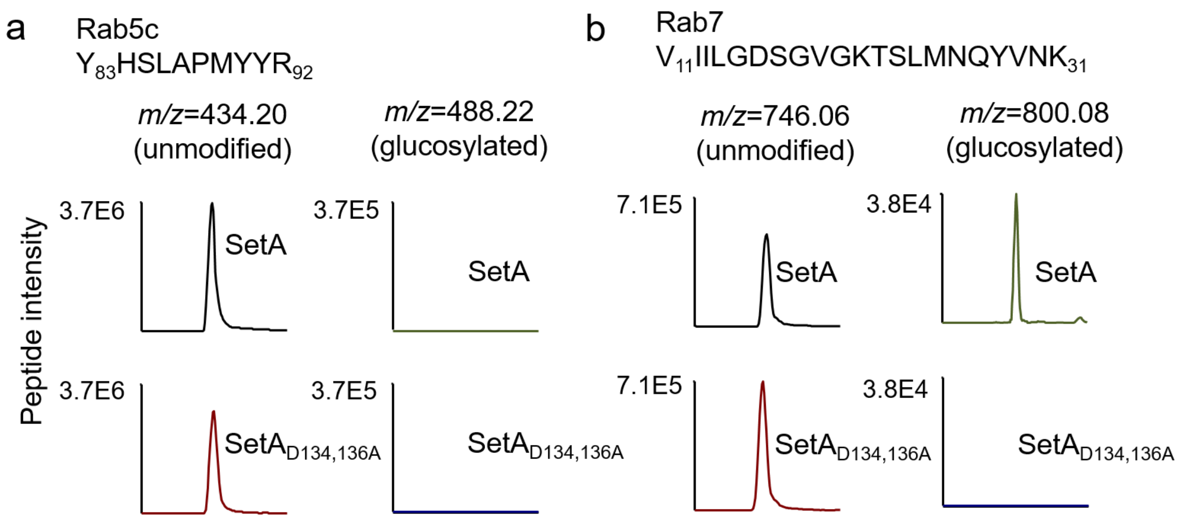

Figure S5. *In vitro* glucosylation assays of Rab5c and Rab7. Equal amounts of purified Rab5c and Rab7 were incubated with either His-SetA or its enzymatically inactive mutant $SetA_{D134,136A}$. Gel-separated Rab5c and Rab7 were digested for further LC-MS/MS analyses. (a) Extracted ion chromatograms of Rab5c peptide Y_{83}HSLAPMYYR_{92} with glucosylation (*m/z* = 488.22) and without modification (*m/z* = 434.20). (b) Extracted ion chromatograms of Rab7 peptide V_{11}IILGDSGVGKTSLMNQYVNK_{31} with glucosylation (*m/z* = 800.08) and without modification (*m/z* = 746.06).

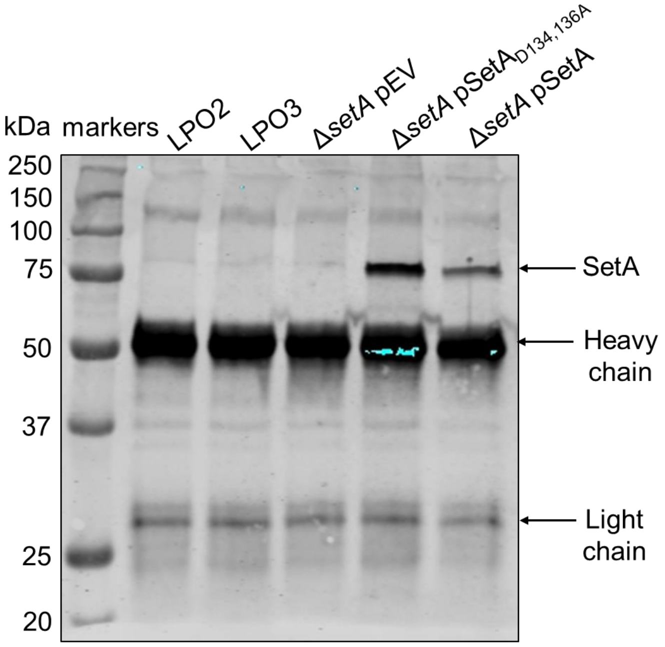

Figure S6. Immunoblotting analyses of translocated SetA during *L. pneumophila* infection. U937 cells were infected by indicated *L. pneumophila* strains at an MOI of 100 for 30 min. SetA was immunoprecipitated from host cytosol using SetA-specific antibodies and were probed by immunoblotting assays.

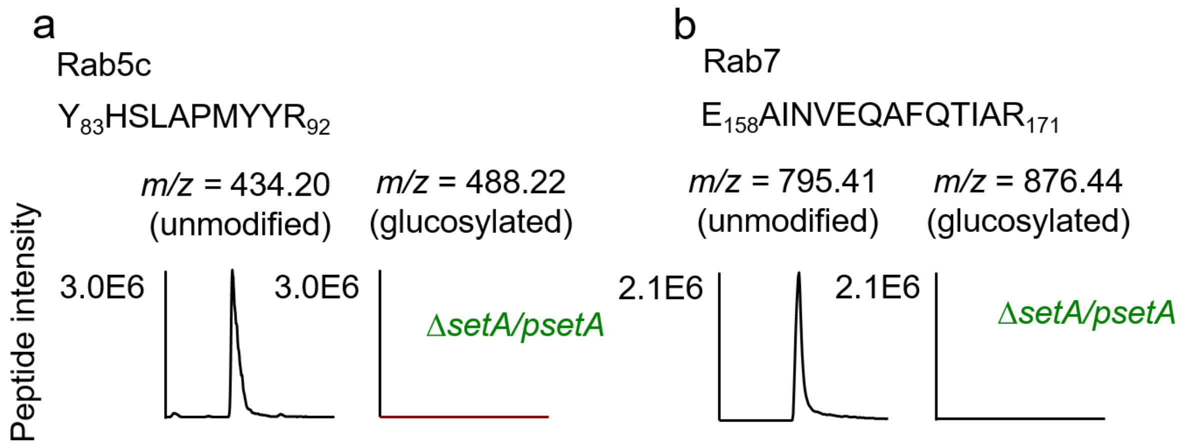

Figure S7. Undetectable levels of Rab5c and Rab7 glucosylation during *L. pneumophila* infection of host cells. (a) The extracted ion chromatograms of Rab5c peptide $Y_{83}HSLAPMYYR_{92}$ are shown. The peak intensities indicate the relative amounts of Rab5c peptides with potential glucosylation (*m/z* = 434.20) or without modification (*m/z* = 488.22). FLAG-tagged Rab5c was isolated from host cells infected by *Legionella* Δ*setA/psetA* strain and analyzed by LC-MS/MS. (b) The extracted ion chromatograms of Rab7 peptide $E_{158}AINVEQAFQTIAR_{171}$ are shown. The peak intensities indicate the relative amounts of Rab7 peptides with potential glucosylation (*m/z* = 876.44) or without modification (*m/z* = 795.41).

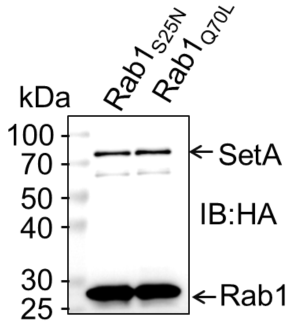

Figure S8. Immunoblotting analyses of SetA co-expressed with $Rab1_{Q70L}$ (GTP-locked Rab1) or $Rab1_{S25N}$ (GDP-locked Rab1) in HEK293T cells.

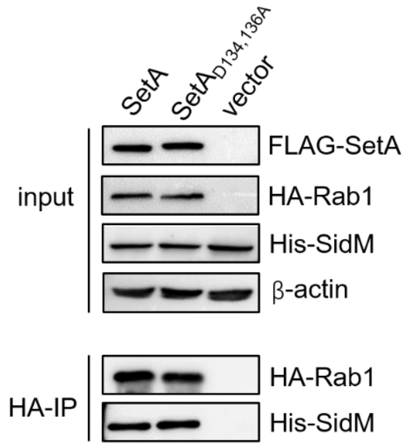

Figure S9. Unaltered binding of SidM to Rab1 upon its glucosylation. HA-tagged Rab1 was co-expressed with SetA or its catalytically inactive mutant SetA$_{D134,136A}$ in HEK293T cells. Samples from HEK293T cells transfected with an empty vector were used as negative controls. The cell lysates were further incubated with purified His$_6$-SidM (2 µg). The binding efficiency of purified His$_6$-SidM to Rab1 was assessed by immunoprecipitation. The levels of Rab1 and precipitated SidM were probed by immunoblotting analyses.

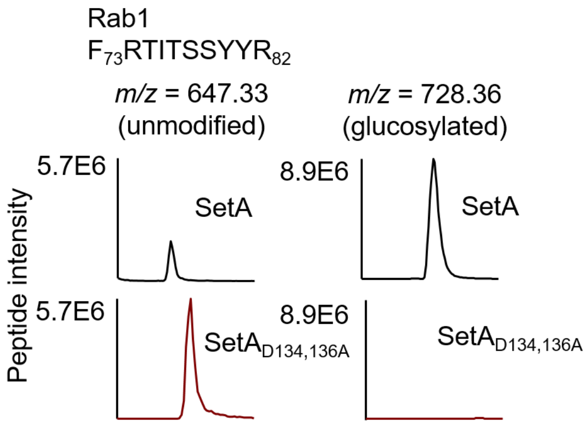

Figure S10. Highly efficient glucosylation of Rab1 upon co-expression with SetA in *E. coli*. GST-Rab1 was purified from BL21 cells expressing His$_6$-SetA or His$_6$-SetA$_{D134,136A}$ and analyzed by LC-MS/MS. The extracted ion chromatograms of Rab1 peptide F$_{73}$RTITSSYYR$_{82}$ are shown. The peak intensities indicate the relative amounts of Rab1 peptides with glucosylation (*m/z* = 728.36) or without modification (*m/z* = 647.33). The ratio of F$_{73}$RTITSSYYR$_{82}$ glucosylation (~75%) was determined by the intensity drop of the unmodified peptide of Rab1 purified from SetA-expressing cells (relative to control samples).

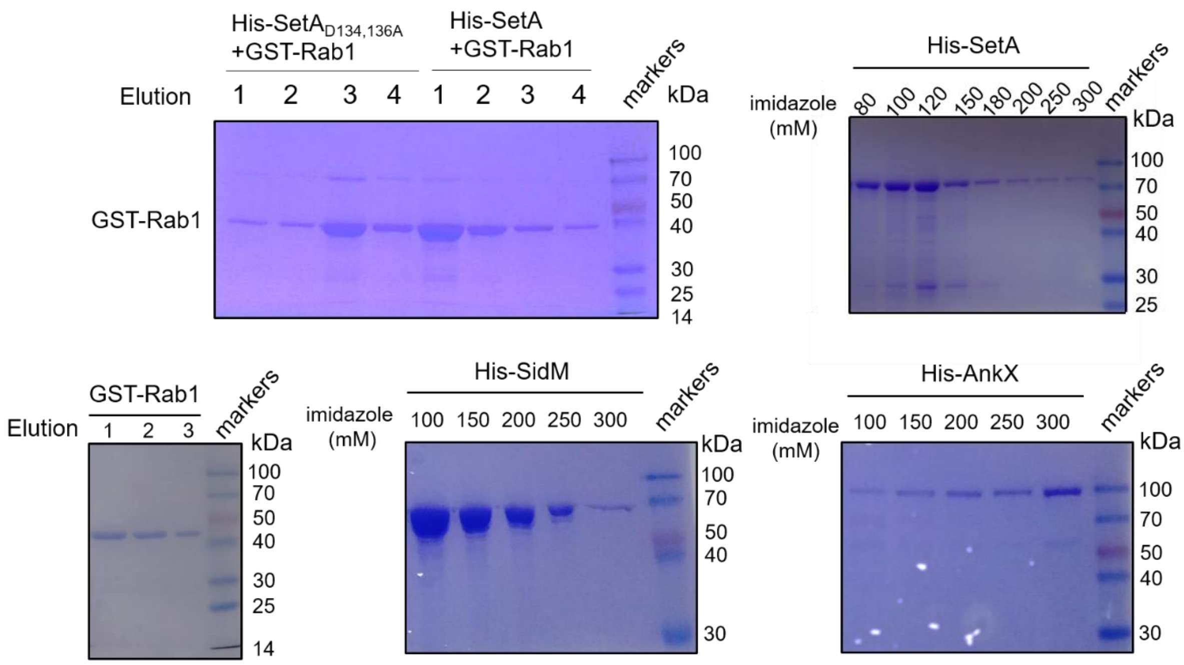

Figure S11. SDS-PAGE analyses of purified proteins used in experiments described in Fig. 5.

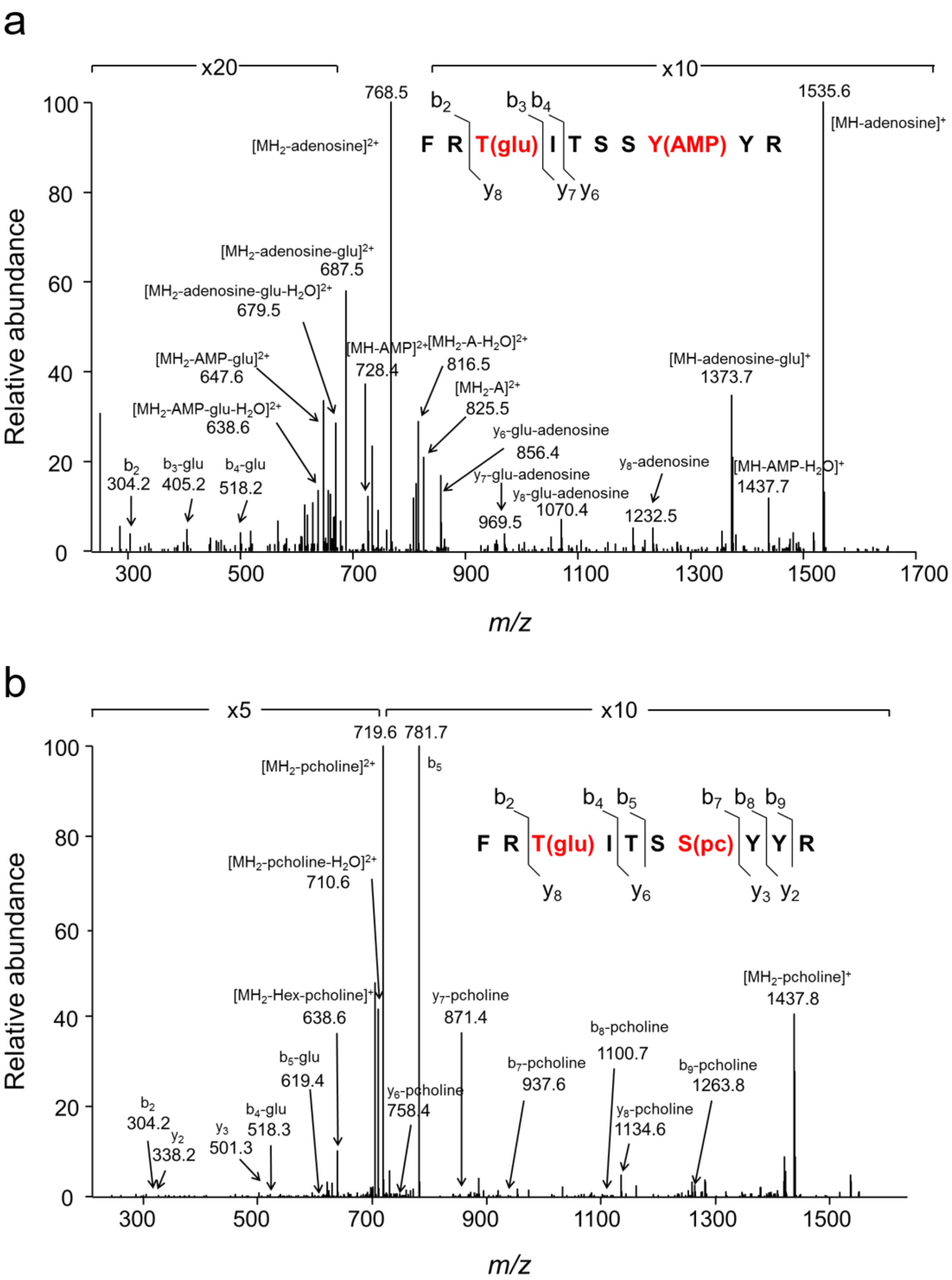

Figure S12. MS/MS spectra of the doubly modified Rab1 peptides that were acquired under collision-induced dissociation (CID). (a) F73RT(glu)ITSSY(AMP)YR82 with both glucosylation and AMPylation. (b) F73RT(glu)ITSSY(pc)YR82 with both glucosylation and phosphorylcholination.

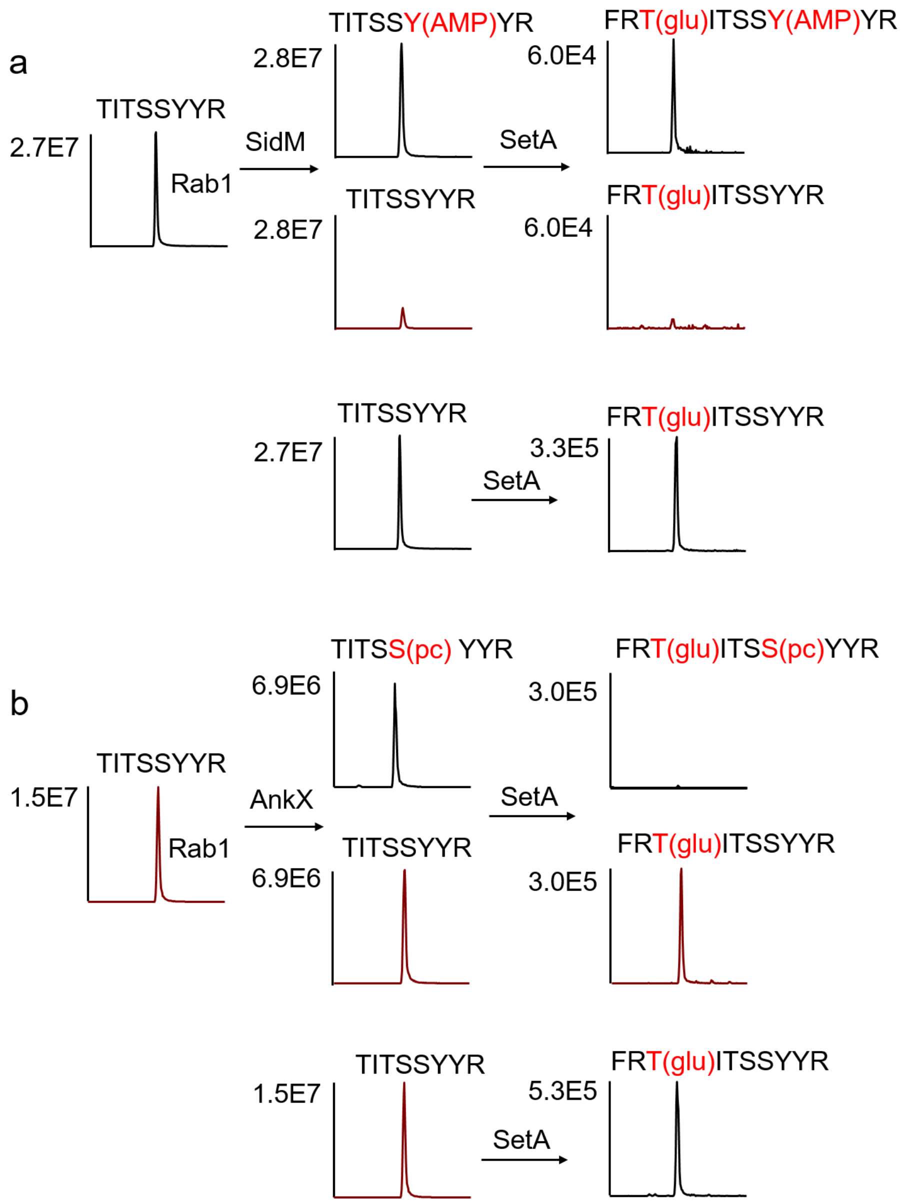

Figure S13. Primary AMPylation or phosphorylcholination of Rab1 adversely impacted subsequent glucosylation. Purified GST-Rab1 was incubated with either SidM or AnkX for potential AMPylation or phosphorylcholination. Half of the reaction mixtures were used for further glucosylation by adding SetA and UDP-glucose. The same amounts of Rab1 were used for control experiments. The modification status of Rab1 was analyzed by LC-MS/MS. The extracted ion chromatograms of different peptides (with or without modifications) are shown. (a) The intensity of Rab1 peptides with primary AMPylation and secondary glucosylation and those peptides with only glucosylation. (b) The intensity of Rab1 peptides with primary phosphorylcholination and secondary glucosylation and those peptides with only glucosylation.
